# Adding propensity scores to pure prediction models fails to improve predictive performance

**DOI:** 10.7717/peerj.123

**Published:** 2013-08-01

**Authors:** Amy S. Nowacki, Brian J. Wells, Changhong Yu, Michael W. Kattan

**Affiliations:** Department of Quantitative Health Sciences, Cleveland Clinic, Cleveland, OH, USA

**Keywords:** Prediction, Propensity score, Calibration curve, Concordance index, Multivariable regression

## Abstract

**Background.** Propensity score usage seems to be growing in popularity leading researchers to question the possible role of propensity scores in prediction modeling, despite the lack of a theoretical rationale. It is suspected that such requests are due to the lack of differentiation regarding the goals of predictive modeling versus causal inference modeling. Therefore, the purpose of this study is to formally examine the effect of propensity scores on predictive performance. Our hypothesis is that a multivariable regression model that adjusts for all covariates will perform as well as or better than those models utilizing propensity scores with respect to model discrimination and calibration.

**Methods.** The most commonly encountered statistical scenarios for medical prediction (logistic and proportional hazards regression) were used to investigate this research question. Random cross-validation was performed 500 times to correct for optimism. The multivariable regression models adjusting for all covariates were compared with models that included adjustment for or weighting with the propensity scores. The methods were compared based on three predictive performance measures: (1) concordance indices; (2) Brier scores; and (3) calibration curves.

**Results.** Multivariable models adjusting for all covariates had the highest average concordance index, the lowest average Brier score, and the best calibration. Propensity score adjustment and inverse probability weighting models without adjustment for all covariates performed worse than full models and failed to improve predictive performance with full covariate adjustment.

**Conclusion.** Propensity score techniques did not improve prediction performance measures beyond multivariable adjustment. Propensity scores are not recommended if the analytical goal is pure prediction modeling.

## Introduction

Propensity score usage seems to be growing in popularity leading researchers to question the possible role of propensity scores in prediction modeling, despite the lack of a theoretical rationale. A number of examples in the medical literature exist (Khanal et al., 2005; Arora et al., 2007; Abdollah et al., 2011); however, it is unknown whether the incorporation of propensity scores was the initial intention of the authors or a response to reviewer requests. Certainly it has been our experience to have grant and manuscript reviewers request the incorporation of propensity scores into prediction focused studies.

It is suspected that such requests are due to the lack of differentiation in observational studies regarding the goals of predictive modeling versus causal inference modeling when a treatment variable is present. In prediction, one aims to obtain outcome prediction estimates that reflect, as closely as possible, observed results. Thus, the goal is to minimize the difference between predicted and observed outcomes. This is in contrast to modeling with a goal of causal inference where one aims to obtain an accurate and precise estimate of the effect of a variable of interest on the outcome. When the variable of interest involves a medical decision (i.e., medication, therapy, surgery), confounding by indication can result in an erroneous conclusion that the variable of interest is in a causal relationship with the outcome by affecting the point estimate, standard error, or both (Vittinghoff et al., 2005). Propensity can be used to minimize residual confounding in non-randomized studies. Such issues are less of a concern for prediction where confounding may not reduce the predictive ability of the model as a whole; they may only affect calculations regarding individual predictors. In other words, a multivariable regression model with confounding may predict accurately, but it may not give valid results concerning any one individual predictor, though the latter may not be of concern to the analyst.

Alternatively, the requests may have more to do with the lack of differentiation between what we term pure prediction modeling and decision prediction modeling. Pure prediction modeling is where the treatment decision has occurred and prediction of future outcome is of primary interest. In contrast are many comparative effectiveness studies where a single model may be utilized for prediction of a patient’s outcome under alternative treatments. We call this decision prediction modeling as the treatment decision has yet to occur and one utilizes the predictive information as part of the decision process. Here the line separating prediction from causal inference is less clear as one aims to minimize the difference between predicted and observed outcomes but also requires good estimation of the treatment effect. It is more conceivable that the incorporation of propensity scores into predictive modeling might be beneficial under these circumstances.

A propensity score is defined as a subject’s probability of receiving a specific treatment conditioned on a set of observed covariates (Rosenbaum & Rubin, 1983). Propensity scores are used to balance observed covariates between subjects from the study groups in order to mimic the situation of a randomized trial (Joffe & Rosenbaum, 1999) and can be used for matching, stratification, or in a regression model as a covariate or weight (Rubin, 1997; D’Agostino, 1998). Because propensity scores are used to address potential confounding by indication, they would not be expected to improve pure prediction, which is not concerned with specific coefficient estimation. Additionally, propensity scores are estimated from regressions that comprise the same covariates included in the traditional prediction models, and only those covariates, thus it would seem mathematically impossible for the propensity scores to add anything – they are simply functions of the same variables already included in the traditional models. Despite this argument, requests for the addition of propensity scores to pure prediction models persist.

Therefore, the objective of this study is to formally examine whether adding propensity scores to a pure prediction model influences prediction performance measures. Our hypothesis is that a multivariable regression model that adjusts for all covariates will perform as well as or better than those models utilizing propensity scores with respect to model discrimination and calibration.

## Materials & Methods

Three published predictive models representing various statistical scenarios motivate the investigation of this research question. We chose to utilize existing datasets instead of doing data simulation because simulation may not represent the type of data encountered in the real world, and most simulated datasets will account for the associations between independent and dependent variables but are not able to mimic the complicated collinearity structures that often exist in real datasets. The three published predictive models are described below.

### Study 1: Surgical Site Infection Prediction (NSQIP)

The objective of this study was to predict organ space surgical site infection (SSI) within 30 days of bowel, colon, or rectal operations (Campos-Lobato et al., 2009). Data for a total of 12,373 major colorectal surgeries were obtained from the American College of Surgeons – National Surgical Quality Improvement Program (NSQIP) database for 2006. A logistic regression model was created using sixteen predictor variables chosen for their association with SSI. The study included two surgical techniques (open vs. laparoscopic) for which selection is heavily influenced by patient characteristics. Hence, this example represents a binomial propensity score scenario within a logistic regression framework.

### Study 2: Renal Graft Failure Prediction (UNOS)

The objective of this study was to predict 5-year graft survival after living donor kidney transplantation (Tiong et al., 2009). Data for a total of 20,085 living donor renal transplant cases were obtained from the United Network for Organ Sharing (UNOS) registry for 2000 to 2003. A Cox proportional hazards regression model was created using eighteen predictor variables chosen for their association with kidney transplantation outcomes. Additionally, a variable representing year of procedure was included as a shift in procedure preference was observed over the four years. The study included two procurement procedures (open vs. laparoscopic) for which selection is heavily influenced by patient characteristics. Hence, this example represents a binomial propensity score scenario within a survival analysis framework.

### Study 3: Diabetic Mortality Prediction (DIABETES)

The objective of this study was to predict the risk of 6-year mortality in patients with type 2 diabetes (Wells et al., 2008). The study was based on a cohort of 33,067 patients with type 2 diabetes identified in the Cleveland Clinic electronic health record that were initially prescribed a single oral hypoglycemic agent between 1998 and 2006 (DIABETES). A Cox proportional hazards regression model was created using twenty-one predictor variables chosen for their association with mortality. The study included patients prescribed one of the four most common types of oral hypoglycemic agents: sulfonylureas (SFUs), meglitinides (MEGs), biguanides (BIGs), or thiazolidinediones (TZDs). It is known that prescribing practice of these medications is associated with patient characteristics. In particular, BIG is often prescribed to the younger “healthier” patients. Hence, this example could represent either a multinomial (SFU vs. MEG vs. BIG vs. TZD) or a binomial (BIG vs. SFU, MEG, & TZD) propensity score scenario within a survival analysis framework.

### Model comparison

Research into variable selection for propensity score models remains active and argues for inclusion of variables that predict treatment assignment only, variables potentially related to the outcome only, or variables associated with both treatment and outcome only (Weitzen et al., 2004; Brookhart et al., 2006; Austin, Grootendorst & Anderson, 2007). We employed the approach of considering variables potentially related to the outcome for inclusion in the propensity score model: the same variables included in the published multivariable models. Once propensity scores are estimated, they can be incorporated into an analysis in one of several ways. This study focuses on the most reasonable approaches for prediction: regression adjustment and weighting. In propensity score regression adjustment, a multivariable regression model is fit that includes the variable of interest (often a treatment) and the propensity score itself, either as a continuous covariate or as a categorical covariate by using the propensity score quintiles as categories. For more than two treatments, the propensity scores of all possible treatments (except the reference treatment) can be included using multinomial regression, or in some cases treatment categories may be combined into a single propensity score (propensity for treatment A versus other) (Imbens, 2000). In inverse probability weighting (IPW), a simple regression model is fit with each observed patient outcome weighted inversely proportional to the conditional probability that he/she would receive the observed choice of treatment given his/her baseline characteristics (aka fitted propensity score) (Rosenbaum, 1987; Robins, Hernan & Brumback, 2000). An IPW estimator “up weights” treated subjects with a low probability of treatment and “down weights” controls that have a high probability of treatment. There is a lack of detailed guidance regarding whether additional variables should be included and if so which additional variables to include in the outcome regression model (D’Agostino & D’Agostino, 2007). D’Agostino & D’Agostino (2007) recommend fitting an outcome model that includes a subset of patient characteristics that are thought to be the most important known potential confounders. Thus, we investigate models that include no additional covariates, select covariates, as well as models that include all covariates for comparison purposes. Table 1 lists all models comprising this investigation and a description of each. Primary comparisons, however, are between the models All, PS and IPW since these models are most commonly employed in the medical literature.

**Table 1:** List of models used for comparison of prediction performance measures.

Model	Description
Naïve	Treatment
All	Treatment
	All covariates
PS	Treatment
	Continuous propensity score
PS quintile	Treatment
	Categorical propensity score
PS + Select	Treatment
	Continuous propensity score
	Select covariates
PS + All	Treatment
	Continuous propensity score
	All covariates
IPW	Treatment
	Inverse probability weighting
IPW + All	Treatment
	Inverse probability weighting
	All covariates
Multi PS	Treatment
	Continuous multinomial propensity scores
Multi PS + All	Treatment
	Continuous multinomial propensity scores
	All covariates
Multi IPW	Treatment
	Multinomial inverse probability weighting
Multi IPW + All	Treatment
	Multinomial inverse probability weighting
	All covariates

### Prediction performance measures

Random 90-10 cross-validation was performed 500 times to correct for optimism in predictive performance measures. With this method, 90% of the data is randomly selected and each of the models fitted. Then, the predictive accuracy is evaluated on the outcomes observed in the remaining 10% subsample. Thus, data used to build a model is never used to assess the predictive accuracy of the model (bias-corrected) (Schumacher, Holländer & Sauerbrei, 1997). Random number seeds were used to select the patients in the training and test dataset to insure that each method was evaluated on identical patients across techniques at each iteration. A calibration curve was created by plotting the quintiles (or maximum number of groups available) of the average predicted probabilities on the observed estimates for the entire cohort. A curve on the 45 degree line represents perfect calibration. The concordance index (i.e., *c* statistic) was used to evaluate model discrimination (Harrell et al., 1982; Harrell, Lee & Mark, 1996). This is defined as the probability that given two randomly selected patients, the patient with the worse outcome was, in fact, predicted to have a worse outcome. Concordance indexes can vary between 0.5 (chance) and 1.0 (perfect prediction). Additionally, the Brier score is reported as a measure of prediction precision (Brier, 1950; Gerds & Schumacher, 2006). The Brier score is a weighted average of the squared differences between the predicted probabilities and the observed outcomes; hence, lower values are better. Each of these prediction performance measures is further described in Steyerberg et al. (2010). Additionally a shrinkage coefficient was obtained to quantify the amount of overfitting for each model (Harrell, Lee & Mark, 1996). The steps of the modeling approach are summarized in Table 2. Statistical analyses were performed using R for Unix, version 2.12.2 with the following packages, rms, Hmisc and pec. There was no external funding source for this study.

**Table 2:** Steps of the modeling approach.

Modeling approach
1. Begin with full dataset.
2. Randomly select 90% of full dataset as Training dataset; remaining 10% of full dataset is Test dataset.
3. Fit propensity model to the Training dataset. Use this model to obtain propensity scores for patients in both the Training and Test datasets.
4. Fit each of the 12 predictive models to the Training dataset.
5. Use model coefficients to obtain predicted probabilities for the Test dataset; do this for each of the 12 predictive models.
6. Calculate prediction performance measures (*c* statistic, Brier score, etc.) on the Test dataset; do this for each of the 12 predictive models.
7. Repeat steps 2–6, 500 times.

## Results and Discussion

**Figure 1:**
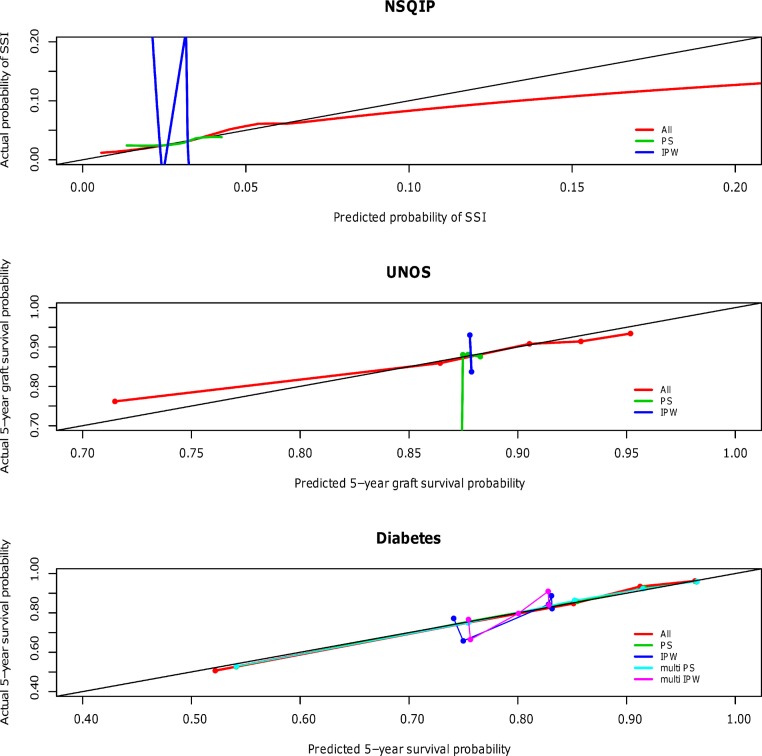
Predictive accuracy by calibration curve among the models in the NSQIP, UNOS and DIABETES studies.

The calibration curves for the NSQIP study show that the published multivariable model adjusting for all covariates most closely fits the diagonal line. Propensity score adjustment and inverse probability weighting performed comparably only when additionally adjusting for all covariates. The weighted propensity analysis using inverse probability treatment weighting (IPW) alone (without adjustment for other variables) model displays substantial over- and underestimation; however, this model is known to have poor properties when the propensity score gets close to zero or one for some observations (i.e., division by numbers close to zero will lead to high variance in the estimator) (Rubin, 2006). Similarly for the UNOS and DIABETES studies, the published regression models that contains all predictor variables (All) outperforms propensity score regression (PS) alone and inverse probability weighting (IPW) alone; performance is relatively comparable when these methods are used in addition to adjustment for all covariates. The calibration curves for all three studies according to model type are shown in Fig. 1 and separated out to illustrate confidence in Appendices A (NSQIP), B (UNOS) and C (DIABETES).

In all three studies, the published multivariable models adjusting for all covariates (All) achieved a higher average concordance index than PS and IPW alone. It is not until these latter two methods also adjust for all covariates that they perform comparably. For each of the three studies, the median and standard error of the concordance indices for all models are reported in Table 3. In summary, the addition of a propensity score affected model discrimination to varying degrees based on the effect of the treatment on the outcome, but did not surpass the published multivariable adjustment model (All) in any scenario. Results were consistent for the Brier scores (data not shown). Multivariable adjustment for all covariates achieved the lowest Brier score while PS and IPW only attained this level of performance when also adjusting for all covariates.

**Table 3:** Discrimination by concordance index and overfitting by shrinkage factor among the models in the NSQIP, UNOS and DIABETES studies.

Study	Performance measure	Naïve	All	PS	PS quintile	PS + Select	PS + All	IPW	IPW + All	Multi PS	Multi PS + All	Multi IPW	Multi IPW + All
NSQIP	median *c*-statistic	0.54	0.66	0.57	0.56	0.57	0.66	0.54	0.64				
	std error	0.001	0.003	0.003	0.003	0.003	0.003	0.001	0.003				
	median shrinkage factor	0.88	0.82	0.84	0.75	0.76	0.82	0.93	0.93				
UNOS	median *c*-statistic	0.50	0.71	0.49	0.49	0.62	0.71	0.48	0.71				
	std error	0.001	0.001	0.001	0.001	0.001	0.001	0.001	0.001				
	median shrinkage factor^*^	−1	0.95	−0.71	−0.37	0.96	0.95	−7	0.98				
Diabetes	median *c*-statistic	0.63	0.75	0.74		0.74	0.75	0.62	0.75	0.74	0.75	0.62	0.75
	std error	0.0008	0.0006	0.0008		0.0006	0.0011	0.0006	0.0008	0.0006	0.0006	0.0008	0.0006
	median shrinkage factor	0.996	0.98	0.998		0.997	0.98	0.99	0.99	0.996	0.98	0.99	0.996

**Notes.**

Shading represents best performing model(s) according to the *c*-statistic.

* Negative shrinkage factors result when treatment variable is poor predictor of outcome and hence a very small likelihood ratio value.

As more complex models typically have better fit, can the improvement in model discrimination be explained by overfitting? The shrinkage factor quantifies the overfitting of a model where values less than 0.85 might be of concern (Harrell, Lee & Mark, 1996) (Table 3). The impact of propensity scores on model overfitting appears to depend on the significance of the treatment and the size of the sample. In the NSQIP study where the treatment effect is impactful and the sample size moderate, there is slight evidence of overfitting with the full multivariable model (All). The impact of propensity scores varies with some alleviating overfit (IPW, IPW + All), some with comparable overfit (PS, PS + All) and others increasing the overfit (PS quintiles, PS + Select). In the UNOS study where the treatment effect is minimal and the sample size is large, there is no evidence of overfitting in the large models containing more parameters. The observed negative values occur when the variable(s) are poor predictors of the outcome resulting in very small likelihood ratio values. Here the shrinkage factor formula is inappropriate and does not provide a valid assessment of model overfit. In the DIABETES study where the treatment effects are impactful and the sample size is large, there is no evidence of overfitting in any model scenario. Thus the superior concordance indices for the multivariable model (All) are not purely a product of overfit models.

Claims have been made that propensity scores improve pure prediction despite lack of theoretical underpinnings (Roberts et al., 2006). This particular investigation, however, focuses on significance of likelihood ratio tests for propensity scores and does not consider commonly accepted measures of predictive performance such as accuracy and discrimination. The results of our study suggest that adjustment for residual confounding using propensity scores does not improve the accuracy of pure prediction models that already include important known predictor variables. This finding held true regardless of the method used for the propensity adjustment (propensity regression versus weighting). These conclusions are not meant to address the potential importance of propensity adjustment when it comes to evaluating the relative impact of individual predictor variables as is done when trying to make causal inferences. Rather, pure prediction models appear not to be affected by residual confounding. These findings are consistent with statistical theory which suggests that confounding may mask the precise point estimates for individual coefficients but should not affect the overall calculated risk when all covariates are considered together.

A limitation of our study is that the results cannot be extrapolated to small sample sizes. While again, there is no theoretical justification for the use of propensity scores in this setting, requests may arise as a perceived benefit of combining multiple variables into one score necessary for model convergence may exist. Another limitation is lack of generalizability in that these results are based on cross-validation and therefore solely reflect reproducibility of the research findings. That is to say that the use of propensity scores does not add value when prediction models are developed and implemented in exactly the same patient population. It is possible, however somewhat unlikely, that propensity scores may improve model performance across different but related patient populations (e.g., populations with different predictor effects).

It seems that propensity adjustments are frequently misunderstood, even by professionals with significant statistical training. Some medical researchers feel that propensity models can completely replace randomized controlled trials by removing all possible confounding by indication. However, the propensity score is only as good as the variables included in its calculation. The propensity score cannot adjust treatment probabilities for unknown or unmeasured factors (Heinze & Jüni, 2011). And, if all known factors are already included in the regression equation then adding additional propensity scores based on those same variables should not and did not improve the overall predicted risk. The present study should simplify risk prediction modeling for researchers, especially as pure prediction modeling increases in popularity. Propensity tools may still be useful in investigations of causal inferences or decision prediction modeling, but they do not play a role in pure prediction modeling with large datasets. In fact, the inclusion of propensity scores may lead to less accurate models by contributing to overfitting, causing an inflation of the variance surrounding the prediction estimate (Rubin, 2001), and leading to extreme variations in estimates for patients at the extremes of the propensity spectrum when using IPW.

## Conclusions

While the use of propensity scores has shown benefit in causal inference modeling, its value in pure prediction has not been empirically demonstrated in these three studies due to its lack of theoretical foundation. The use of propensity scores did not improve prediction performance measures; whereas adjusting for all covariates in the model resulted in better predictive performance. Thus, careful consideration of the modeling goal must be incorporated into the choice to use propensity score techniques. Propensity scores are not recommended if the analytical goal is pure prediction modeling.

## Supplemental Information

10.7717/peerj.123/supp-1 Supplemental Information 1 Predictive accuracy by calibration curve with confidence among the models in the NSQIP study.

10.7717/peerj.123/supp-2 Supplemental Information 2 Predictive accuracy by calibration curve with confidence among the models in the UNOS study.

10.7717/peerj.123/supp-3 Supplemental Information 3 Predictive accuracy by calibration curve with confidence among the models in the DIABETES study.
